# Interacting with autistic virtual characters: intrapersonal synchrony of nonverbal behavior affects participants’ perception

**DOI:** 10.1007/s00406-023-01750-3

**Published:** 2024-01-25

**Authors:** Carola Bloch, Ralf Tepest, Sevim Koeroglu, Kyra Feikes, Mathis Jording, Kai Vogeley, Christine M. Falter-Wagner

**Affiliations:** 1 Department of Psychiatry and Psychotherapy, Medical Faculty, LMU Clinic, Ludwig-Maximilians-University, 80336 Munich, Germany; 2 Department of Psychiatry and Psychotherapy, Faculty of Medicine and University Hospital Cologne, University of Cologne, 50937 Cologne, Germany; 3 Cognitive Neuroscience, Institute of Neuroscience and Medicine (INM-3), Forschungszentrum Juelich, 52425 Juelich, Germany

**Keywords:** Autism spectrum disorder, Interaction, Nonverbal, Gaze, Gestures, Joint attention

## Abstract

Temporal coordination of communicative behavior is not only located between but also within interaction partners (e.g., gaze and gestures). This intrapersonal synchrony (IaPS) is assumed to constitute interpersonal alignment. Studies show systematic variations in IaPS in individuals with autism, which may affect the degree of interpersonal temporal coordination. In the current study, we reversed the approach and mapped the measured nonverbal behavior of interactants with and without ASD from a previous study onto virtual characters to study the effects of the differential IaPS on observers (*N* = 68), both with and without ASD (crossed design). During a communication task with both characters, who indicated targets with gaze and delayed pointing gestures, we measured response times, gaze behavior, and post hoc impression formation. Results show that character behavior indicative of ASD resulted in overall enlarged decoding times in observers and this effect was even pronounced in observers with ASD. A classification of observer’s gaze types indicated differentiated decoding strategies. Whereas non-autistic observers presented with a rather consistent eyes-focused strategy associated with efficient and fast responses, observers with ASD presented with highly variable decoding strategies. In contrast to communication efficiency, impression formation was not influenced by IaPS. The results underline the importance of timing differences in both production and perception processes during multimodal nonverbal communication in interactants with and without ASD. In essence, the current findings locate the manifestation of reduced reciprocity in autism not merely in the person, but in the interactional dynamics of dyads.

**Supplementary Information:**

The online version contains supplementary material available at 10.1007/s00406-023-01750-3.

## Introduction

Temporal structures that emerge in social interactions between individuals constitute a shared rhythm that influences the mutual evaluation and success of the interaction [1–6]. However, temporal structures already occur at the subpersonal level as *intrapersonal synchrony* (IaPS) that can be defined as the intrapersonal temporal coordination of multimodal signals (e.g., gaze, gestures, facial expressions, speech) within individuals while engaged in interactions [7]. It is assumed that IaPS is a pre-requisite for interpersonal alignment [7, 8]. IaPS includes implicit processes acquired from infancy on through, for example, behavioral imitation and time-sensitive communication structures in caregiver–child interactions [4, 9–14].

**Fig. 1:**
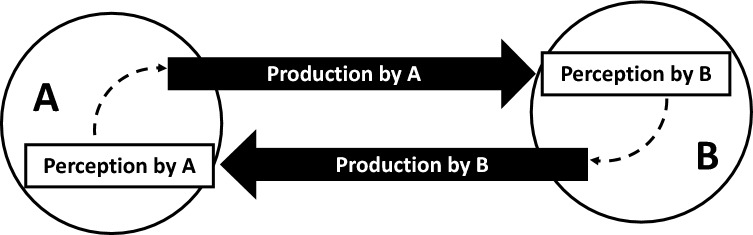
Simplistic illustration of intrapersonal synchrony (IaPS) production and perception in a dyadic setting. Two individuals **A** and **B** are schematically displayed as circles. The production of intrapersonally synchronized behavior is perceived by the interaction partner. The dashed lines indicate the assumption that the perception systematically affects response behavior within the respective observer who will also produce multimodal communicative signals. A simplistic reciprocal interaction sequence could be regarded as a circular process in which production on side (**A)** influences perception and subsequently production on side (**B)**, which in turn influences perception and production on side (**A**)

Considering IaPS in an interpersonal scenario, *production* and *perception* of IaPS have to be distinguished, see simplistic scheme in Fig. 1. The intrapersonal coordination of multimodal signals will influence, which and how series of multimodal signals are perceived as a signal-unit through temporal coherence of signal expressions (e.g., as operationalized in the current study, gaze and pointing gesture coupling). The interaction partner can then in turn respond effectively to these signal-units. Thus, an interaction sequence could be conceived as a circular process in which the production of IaPS depends on its perception in the counterpart and vice versa.

Autism spectrum disorder (ASD) is a pervasive developmental disorder that is being clinically diagnosed on the basis of various criteria including characteristics in nonverbal communication and social interactions [15]. Studies have shown reduced interpersonal temporal alignment during dyadic interactions of individuals with and without ASD [16–18]. On the individual level, deviations in the production of IaPS are reflected by diagnostic items in the ADOS [19] and by studies that reported increased and more variable delays in the intrapersonal production of multimodal signals [20, 21]. These differences in IaPS production could potentially affect communication dynamics and explain why interactions between individuals with and without ASD are temporally less aligned and are judged less favorable by typically developed (TD) individuals [20, 22–26]. Having identified systematic group differences in the production of IaPS [20, 21, 27] and assuming that the production of IaPS will affect both perception and production (response behavior) in interactants (see Fig. 1), we systematically investigate the communicative effects of ASD-specific differences in the production of IaPS.

Considering the perception of IaPS, it is plausible to assume perceptual automatisms acquired from infancy [28–32]. In this context of highly automatized communication behaviors, deixis (i.e., making a spatial reference from a personal perspective [32, 33] and the more complex multimodal establishment of *joint attention* through deictic signals (i.e., gaze and pointing gestures) are of particular interest. Infants and caregivers already use this type of communication behaviors before any verbal exchange to spatially reference objects to each other [34, 35]. Deictic actions subserve the establishment of joint attention [33] and are central for language acquisition in infants [32, 36] as well as for the development of social cognition [37, 38]. Indeed, atypical perception of joint attention and atypical response behavior during joint attention sequences have been frequently reported in ASD samples [30, 39–42].

To our knowledge, it has not been investigated if and how different IaPS production levels during multimodal joint attention affect observers. In a virtual interaction paradigm, Caruana et al. [43] showed that gaze movements that are congruent with a pointing gesture are automatically integrated in TD observer’s perceptual process and lead to faster responses. Whether and how attenuated gaze–gesture coupling affects responses in observers is unknown. Furthermore, it remains elusive whether there might be an in-group advantage of observers with ASD when observing gaze-gesture coordination that is prototypical of the production in ASD [21].

## Study aims

The aim of the current study was to quantify the effects of differences in gaze-pointing delays (i.e., IaPS production levels) on communicative efficiency in a crossed design with ASD and TD observers. This design allowed to study “in-group” and “out-group” effects referring to the two observer groups. IaPS production levels were based on original data from a prior study [21]. The real-life parameters from the previous study were mapped onto two virtual characters in the current study. In a virtual communication task, participants had to decode the nonverbal behavior displayed by the virtual characters and respond to it. Effects of different IaPS production levels on three perceptual domains were measured, namely (i) decoding speed (response times), (ii) visual information search behavior (gaze recordings), and (ii) post hoc impression formation. We assumed that gaze–gesture delays as produced by TD individuals would lead to effective and thus fast signal decoding in TD observers and that signal encodings outside the TD range would slow down response times and negatively affect post-hoc impression formation ratings. Furthermore, we assumed that these effects would be diminished for observers with ASD when observing IaPS levels as produced by TD individuals.

## Methods

### Participants

Inclusion criteria were age between 18 and 60 years and normal or corrected to normal vision. An ASD diagnosis (F84.5 or F84.0 according to ICD-10 [15]) was a mandatory inclusion criterion for the ASD group. Depression and use of antidepressants were no exclusion criteria for the ASD group because of the high co-occurrence of depression in ASD and our aim to recruit a representative sample [44]. Beyond that, any current or past psychiatric or neurological disorder was an exclusion criterion as well as current intake of psychoactive medication. All inclusion criteria were preregistered. ASD participants were recruited via the outpatient clinic for autism in adulthood at the University Hospital Cologne. ASD diagnosis was provided by two independent clinicians according to the German national health guidelines for ASD diagnostics [45]. TD participants were recruited as age- (± 5 years) and gender-matched pairs via social media and the hospital’s intranet.

Prior to study realization, a power analysis was run in G*Power [46] for sample size planning. Aiming to obtain a power of 0.95 to detect an effect size f^2^ of 0.35, we planned to recruit 25 individuals per group. Due to a technical issue, nine participants with ASD and eight TD participants were unintentionally recorded with missing trials (see Sect. “Data preprocessing”). After fixing the problem, we decided to recruit 10 more individuals per group and subsequently test whether the technical issue had affected the results. In total, we tested 36 participants with ASD from which one person’s data had to be excluded from final analysis due to their high diopter having interfered with the eye-tracking system. One further person with ASD was excluded who had too many missing responses (49.4% of response data missing).

The final sample included 34 individuals with confirmed ASD diagnosis (22 identified as male, 11 as female, one as diverse) and 34 TD individuals (23 identified as male, 11 as female). All individuals in the ASD group who enrolled for testing had a confirmed F84.5 diagnosis. All participants provided written informed consent prior to testing and received financial compensation for their participation. Sample characteristics and group comparisons on neuropsychological and autism-screening scales are depicted in Table 1.

**Table 1:** Sample characteristics and group comparisons

	TD		ASD		Statistic	*p*
	*M*	*SD*	*M*	*SD*		
Age^B^	37.77	12.73	39.52	12.80	*U* = 618	0.630
AQ^B^	13.39	5.89	39.59	5.77	*U* = 1116	< 0.001
EQ^B^	47.06	13.77	15.41	8.09	*U* = 32	< 0.001
SQ^A^	26.35	10.66	39.18	15.08	*t*(59.4) = 4.05	< 0.001
SPQ	55.15	14.56	53.50	15.06	*t*(66) = – 0.459	0.648
ADC^B^	17.41	9.39	51.32	14.80	*U* = 1126	< 0.001
BDI-II^B^	4.79	5.70	15.77	11.48	*U* = 928	< 0.001
D2	104.35	11.05	109.44	12.19	*t*(66) = 1.80	0.076
vIQ	107.03	8.88	109.88	11.39	*t*(65) = 1.14	0.258

*AQ* Autism Spectrum Quotient, *EQ*  Empathy Quotient, *SQ*  Systemizing Quotient, *SPQ*  Sensory Perception Quotient, *ADC*  Adult Dyspraxia Checklist, *BDI*  Beck’s Depression Inventory II, *D2*  test of concentration abilities, *vIQ*  verbal Intelligence Quotient assessed by ‘Wortschatztest’

Results of Student ‘s *t*-tests with *α* = 0.05.

^A^Unequal variances indicated by Bartlett tests and Welch approximation of *df* applied

^B^Violation of normality indicated by Shapiros test and results of Wilcoxon *U*-tests reported

AQ data and vIQ data missing for one TD participant each

### Study protocol

The study protocol was approved by the ethics review board of the medical faculty at the University of Cologne (case number: 16–126) and preregistered in the German register for clinical studies (reference number: DRKS00011271) and the Open Science Framework (OSF) (osf.io/dt6vh). The study was performed in line with the principles of the Declaration of Helsinki.

Prior to the experiment, participants completed German versions of the Autism Spectrum Quotient [47], Empathy Quotient [48], Systemizing Quotient [49], Sensory Perception Quotient [50], and Adults Dyspraxia Checklist [51] as standard neuropsychological testing in the autism outpatient clinic. Testing began with a brief introduction and obtaining written informed consent. Demographic data were collected and the Beck’s Depression Inventory [52] was administered. Participants then performed the virtual communication task and answered a post-hoc questionnaire after the task. Afterward they performed a perceptual simultaneity task, which is reported here for completeness but not discussed further. Finally, the ‘Wortschatztest’ [53] and the D2 [54] were administered. 40 participants (24 with ASD) participated in another study on social interactions and person judgment in ASD on the same day, with a break between studies. The order of the studies was randomized and counterbalanced to prevent systematic order effects.

### Animation

Two virtual characters were pre-selected by a pilot study (see Supplementary Material 1 and Supplementary Table 1). Animation of the virtual characters was conducted in Autodesk ® Motion Builder in which spatial and temporal parameters from real behavioral parameters in a previous study [21] were closely implemented. In this prior study, participants’ task was to communicate the position of a target stimulus that appeared on the left or right side of an opposite screen to an experimenter using both gaze and pointing gestures [21]. For specific information on animation, see Supplementary Material 2.

### Virtual communication task design

The virtual communication task consisted of 108 interaction sequences in 6 blocks of 18 trials each. The 6 blocks were divided into 3 blocks per character and condition. All participants interacted with both characters and observed both IaPS conditions. The two characters prototypically displayed different IaPS levels: One displayed a temporal coordination of gaze and gestures that was based on measures in TD individuals (IaPS_TD_) and the other displayed behavior measured in individuals with ASD (IaPS_ASD_). The assignment of the two virtual characters to one of the IaPS_TD_ or IaPS_ASD_ conditions and the order in which participants interacted with the two characters was randomized and counterbalanced within groups.

In each trial, the characters selected one of two items located on screens available in the scenery on the left and right side. The preferred item was communicated by a gaze shift and a pointing gesture similar to a joint attention sequence. Participants were instructed to quickly and accurately select the indicated object by keypress, once they knew what their partner had selected. Participants were not informed in advance that the virtual characters were prototypical of the production modes of the two groups (ASD and TD).

### Trial procedure and experimental manipulation of IaPS

**Fig. 2:**
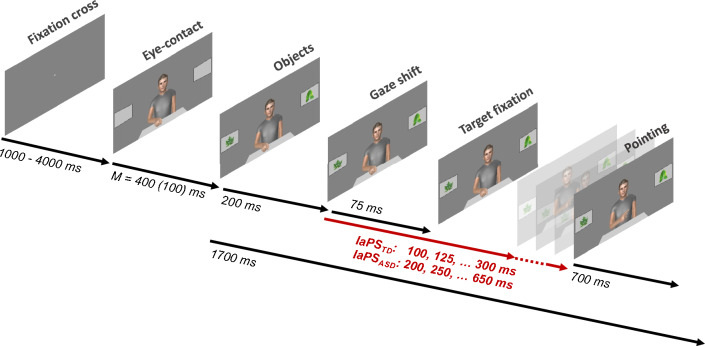
Trial procedure during virtual communication task. Each trial started with a fixation cross displayed for an average duration of 2500 ms (± 1500 ms, uniformly distributed). The virtual character appeared, facing participants, for an average duration of 400 ms (SD = 100 ms, normally distributed). The objects appeared and after a latency of 200 ms a gaze shift (lasting 75 ms) occurred toward one of the two objects. The respective object was then fixated until the end of the trial. The pointing gesture onset occurred after the gaze shift onset with a variable delay, that accounted for the IaPS conditions (see red arrow). The pointing gesture itself took 700 ms from onset to peak. From object appearance until the end, trials had a fixed duration of 1700 ms

The trial procedure of the virtual communication task is depicted in Fig. 2. Each trial started with a fixation cross that was displayed for an average duration of 2500 ms (± 1500 ms, uniformly distributed). Then the characters appeared with the hand resting on the table and gazing toward the observer. This eye-contact position of the character was held for an average duration of 400 ms (SD = 100, normally distributed). The temporal variability in these initial trial sections was implemented to bring a temporal dynamic to the communication task. Next, an object appeared on the left and another on the right screen respectively. After a duration of 200 ms, the character “chose an object” and indicated the preferred object with deictic nonverbal signals to the observer. Thus, a gaze shift to one of the two objects with a duration of 75 ms occurred. The pointing gesture started after a variable delay after the gaze shift onset: In the IaPS_TD_ condition, temporal delays ranged from 100 to 300 ms in steps of 25 ms. This resulted in nine temporal conditions (100, 125, 150, 175, 200, 225, 250, 275, 300 ms) that were displayed once per side (left, right) in a randomized order per block. In the IaPS_ASD_ condition, temporal delays ranged from 250 to 650 ms in steps of 50 ms. This resulted in eight temporal conditions (250, 300, 350, 400, 450, 500, 550, 600, and 650 ms) that were displayed once per side (left, right) in a randomized order per block. The pointing gesture had a duration of 700 ms from onset to final linger position. The trial ended with the character looking and pointing to the target object. Overall, each trial had a fixed duration of 1700 ms from the appearance of the objects to the disappearance of the character and the start of a new trial.

### Apparatus & stimuli

The experiment was conducted in a windowless room with steady illumination. The experimental script was run in PsychoPy [55], running on a HP desktop computer. Stimuli were presented on an ASUS 27 inch LCD widescreen monitor with a resolution of 2560 × 1440 pixels and a refresh rate of 120 fps. An EyeLink 1000 Plus eye-tracker by SR Research® in a desktop-mounted mode recorded participants monocular gaze movements with 1000 fps temporal resolution. Gaze recordings were adjusted to the screen using a 9-point calibration procedure. Participants’ heads were positioned on a chin-rest (SR Research®) that also stabilized the forehead in a distance of 94 cm from the edge of the table to the screen. A keyboard (German layout) was positioned in front of participants so that the V key was centered in front of the chin rest. Subjects were instructed to place the index finger of the left hand on the Y key and the index finger of the right hand on the M key. Once the virtual partner had selected an object, participants were to press one of the two keys (Y key for left object, M key for right object) in order to select the chosen object for their interaction partners. The time of the key press (measured from object appearance, see Fig. 2) was additionally logged in the gaze data using functions provided by the PyLink Module (SR Research®).

The stimuli used in the study consisted of videos of the animated characters (see Sect. “Animation”). The objects were chosen from an object library provided by Konkle et al. [56]. They appeared on the two screens (left and right) and were derived from the same object category (e.g., butterflies) that had a similar appearance in color and form. The two screen frames were drawn in a way that the same perspective was obtained symmetrically on both sides.

### Impression formation ratings

After the virtual communication task, participants were asked to rate each character with regard to nine items about impression formation [24] on a polar slider scale. Items included clumsiness, likeability, involvement, uncertainty, strangeness of communication, clarity of communication, willingness to spend time with this person, willingness to have a conversation with this person, and willingness to ask this person for help. The order of rating items was randomized and the order of characters was randomized and counterbalanced between participants.

### Data preprocessing

#### Response time data

The time of the key press from the appearance of the objects was recorded. Response times thus indicated the time at which participants decoded the nonverbal communication by their virtual partner. Due to a technical error, one video of one character (rightward gaze-gesture delay of 350 ms) was not presented. This resulted in the systematic loss of three trials (one per block) for 17 participants (9 ASD, 8 TD) in the IaPS_ASD_ condition. We corrected this issue and subsequent participants were tested with the corrected setup. A binary variable in the subsequent analysis was used to dummy-code participants tested before or after correction (see Table 2, model 5).

**Table 2:**
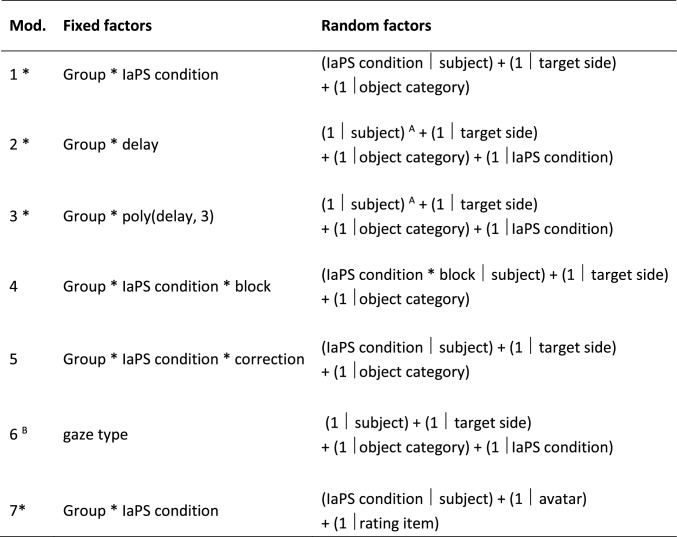
Linear mixed effects models predicting response times (models 1 – 6) and virtual character ratings (model 7) with fixed and random factors

*preregistered on OSF (https://osf.io/dt6vh)

^A^Including random slopes for delays resulted in a convergence failure

^B^This model was calculated separately for both groups because of the potential confounding of group and gaze type

All models were fit using the BOBYQA optimizer. Model compositions are given in *lme4* grammar [60]

Trials with missing responses were excluded. In the TD group, there were *n* = 48 (1.3%) missing responses in total (range 1–7 in 18 subjects) and in the ASD group, there were *n* = 132 (3.6%) missing responses (range 1–22 in 29 subjects). Furthermore, trials with > 1 keypresses were excluded. In the TD group, there were *n* = 39 (1.1%) trials with multiple responses (range 1–18 in 14 subjects). In the ASD group, there were *n* = 31 (0.9%) trials with multiple responses (range 1–12 in 11 subjects). Of a maximum of 108 trials, 81–108 trials per person were analyzed in the TD group and 86–108 trials per person were analyzed in the ASD group.

#### Gaze data

For the gaze data, only the previously mentioned missing trials (see Sect. “Response time data”) were excluded. Gaze recordings started with object appearance and regions of interest (RoI) were set for the virtual characters eye area (RoI *gaze*), their gesture trajectory (RoI *gesture*), and the area located between the gaze and gesture areas (RoI *upperBody*). Additionally, the two object areas were defined trial-wise as a target-object area (RoI *target*), and a distractor-object area (RoI *distractor*). Gaze events (saccade onsets/offsets; fixation starts/ends) were read out from the sample data using the online parser provided by EyeLink that applied a velocity threshold for saccades of 30°/second. Dwell times on each predefined RoI were read out per trial using the software Dataviewer provided by SR Research®. Dwell times in regions other than those defined (i.e., random regions) were excluded. After that, relative RoI dwell times were calculated for each IaPS condition and each subject as the percentage of dwell time on the respective RoI relative to the sum of dwell times in all RoI. These percentages of dwell times in RoIs per IaPS condition were used to classify individuals into gaze types (see Supplementary Material 3 for specifics on the gaze type classification).

#### Post hoc impression formation

The post hoc impression formation judgements were made on a semi-continuous slider scale ranging from 0 to 100 in single steps. For three rating items (clumsiness; strangeness of communication; insecurity), ratings were rescaled so that high values represented positive valence (e.g., less clumsy; less strange; less insecure) and low values indicated negative valence.

#### Statistical analysis

Data analysis was conducted in RStudio version 1.4.1103 [57] using the R language version 4.0.3 [58] and functionalities of the *tidyverse* package library [59].

#### Mixed effects models

Mixed effects models were fitted and analyzed using the packages *lme4* [60] and *afex* [61], see Table 2.

Model assumptions (no multicollinearity, homoscedasticity, and normality of residuals) were visually inspected with functions included in the *performance* package [62]. Likelihood ratio tests (LRT) of nested model were conducted in order to infer if a fixed factor improved model fit above chance level against an alpha of 0.05 using the *afex* function mixed(). Type-III sum of squares was used for all tests. Sum coding was applied to all factors and continuous factors were scaled and centered before implementation in the model in order to retain interpretable main and interaction effects [63, 64].

## Results

### Decoding speed

**Fig. 3:**
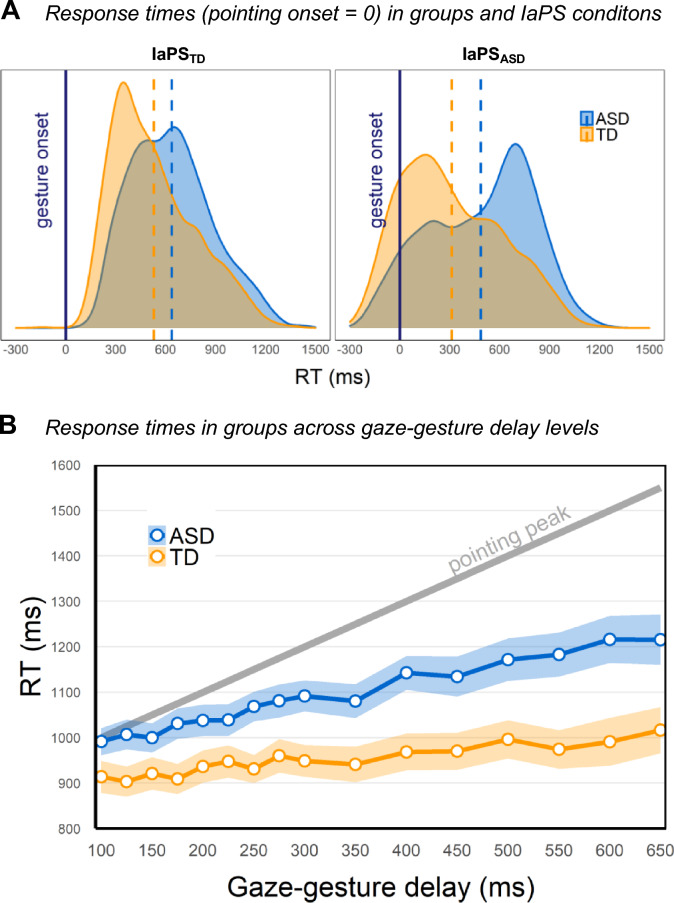
Decoding speeds at varying intrapersonal synchrony (IaPS) levels. **A** Raw response times are displayed centered to the pointing gesture onsets of virtual characters (= zero *x*-axis intercept) for illustration purposes. Responses to the character displaying gaze-gesture delays prototypical of TD (IaPS_TD_) are displayed in the left panel; responses to gaze–gesture coupling prototypical of ASD (IaPS_ASD_) are displayed in the right panel. TD observer group (orange) and ASD observer group (blue). Dashed lines represent observer group means. Negative response times indicate that observers responded before the virtual character started the pointing gesture. **B** Response times averaged across gaze–gesture delay levels (*x*-axis) as white dots per observer group (TD group in orange; ASD group in blue). Average response times were calculated per delay level independent of the nesting in IaPS condition (e.g., delay of 250 ms was equally present in IaPS_TD_ and IaPS_ASD_ and the mean for this delay was calculated by pooling values over IaPS conditions). Ribbons show standard errors of the marginal means. Gray diagonal line represents the point at which the characters pointing gestures peaked (i.e., reached steady linger position with the extended index finger pointing toward target) relative to each delay condition.

Response times in both observer groups and IaPS conditions are depicted in Fig. 3A, where the response times have been centered on the start of the virtual character pointing gestures for illustration purposes. In the TD group, responses in the IaPS_ASD_ condition (Fig. 3A, right panel) partly even preceded gesture onsets which was less the case for observers with ASD. In line with that, LRTs of coefficients in Model 1 (see Table 2) showed that there was a significant effect of IaPS condition, with slower responses for characters displaying the IaPS_ASD_ condition (*χ*^2^(1) = 13.43, *p* < 0.001). This IaPS effect was enlarged for individuals with ASD, as indicated by a marginally significant interaction term (*χ*^2^(1) = 3.85, *p* = 0.050), which contradicts a putative in-group advantage. Additionally, there were overall slower decoding speeds in the ASD group (*χ*^2^(1) = 7.85, *p* = 0.005). Current symptoms of depression, indicated by BDI-II scores, did not significantly affect decoding speed (*χ*^2^(1) = 2.15, *p* = 0.142) neither did the interaction of BDI-II scores with group (*χ*^2^(1) = 0.99, *p* = 0.321).

Decoding speeds across different gaze–gesture delays are depicted in Fig. 3B. Since gaze was always the primary signal, a flat line in these graphs would have suggested that observers consistently responded based on the gaze signal only and that varying the delay of the subsequent gesture had no effect. Conversely, a line parallel to the gray guide line in Fig. 3B (annotated ‘pointing peak’) would have suggested that a particular gesture event (e.g., peak of pointing gesture) was consistently used as a decision anchor across delay levels. Any other trajectory indicates that, on a group level, responses were not made based on unimodal signal anchors only, but rather on some form of integration of gaze and gesture signals. In fact, the trajectories in both observer groups indicate integration of signals during decoding, with response times in both groups increasing more or less across delay levels. LRTs (see Model 2 in Table 2) showed a considerable effect of delay levels (*χ*^2^(1) = 233.09, *p* < 0.001) that was more pronounced in the ASD group (interaction group * delays; *χ*^2^(1) = 86.63, *p* < 0.001). Including a polynomial term (see Table 2 Model 3) did not improve the model fit (comparison of Model 2 and Model 3, see Table 2) (*χ*^2^(4) = 4.93, *p* = 0.294). The increase of response times with IaPS delays thus rather presented as a gradually increasing linear relationship without a specific delay at which decoding speeds were particularly faster or slower. The linear increase was however steeper in the ASD group, as indicated by the significant interaction.

As a next step, the influence of experimental blocks was included in the models in order to investigate possible training effects in both observer groups. Response times decreased over blocks (see Supplementary Fig. 1). LRTs (see Table 2 Model 4) indicated that this was a significant effect (*χ*^2^(1) = 42.99, *p* < 0.001) with no significant difference between groups (i.e., non-significant two-way interaction term group * block; *χ*^2^(1) = 0.04, *p* = 0.838). Group, condition, and group * condition effects remained significant with the inclusion of block (see Supplementary Table 2 for model estimates). Thus, there appears to have been a training effect, with observers responding faster over time, but this effect was equally pronounced in observers with and without ASD. Hence, the difference between the groups in the effect of IaPS levels cannot be ascribed to a difference in training.

The technical error (i.e., missing condition for some participants, see Sect. “Data preprocessing”) did not significantly affect response times, as including the binary correction variable (see Table 2 Model 5) did not improve model fit above chance level (*χ*^2^(1) = 0.11, *p* = 0.745). Group and IaPS effects remained significant, only the interaction term in this model was slightly above significance threshold (*χ*^2^(1) = 3.64, *p* = 0.056), possibly due to decreased power in the model with more predictors (see Supplementary Table 3 for model estimates).

### Exploratory gaze analysis

**Fig. 4:**
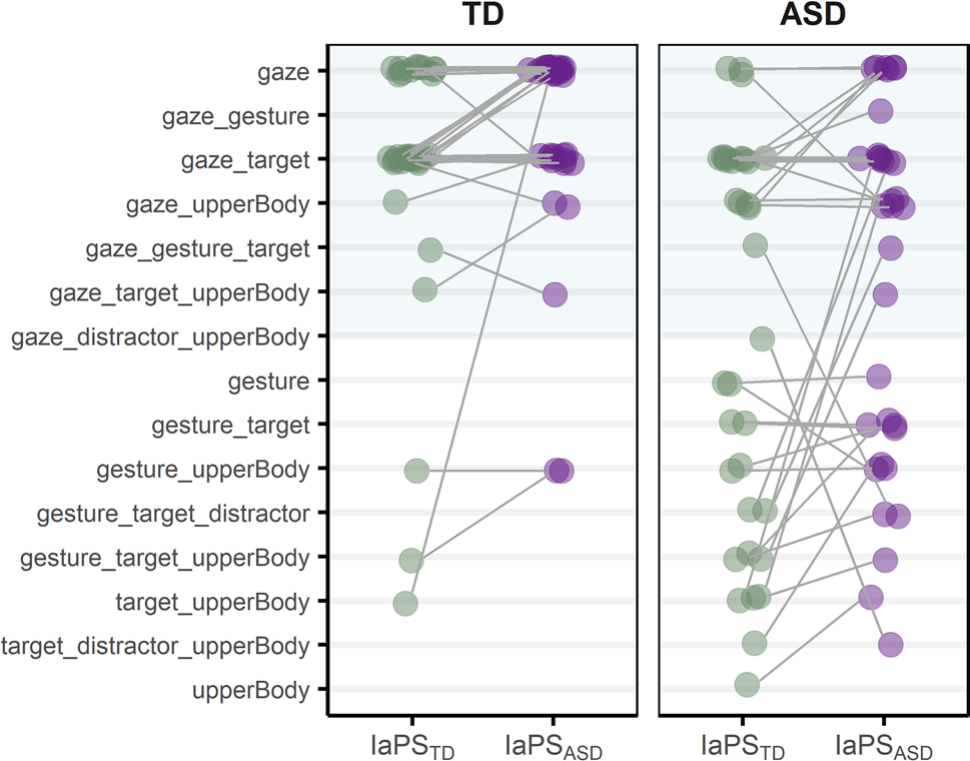
Gaze types (y-axis) in groups of observers (TD, ASD) and IaPS conditions (IaPD_TD_, IaPS_ASD_). Dots display gaze types in IaPS conditions for each subject (IaPS_TD_ left; IaPS_ASD_ right) in both groups of observers (TD left panel; ASD right panel). Two dots per participant, one in each IaPS condition, are connected with gray lines. Horizontally aligned gray lines show individuals whose gaze type did not vary across conditions, vertical lines show individuals whose gaze type varied with IaPS condition. Light blue area in the upper parts of both panels highlight gaze types that included the characters eye region

Summary statistic and group comparisons of the gaze recordings are reported in Table 3. Individuals with and without ASD differed significantly in how much they fixated gaze, gesture, and upper body regions. Gaze types in groups and IaPS conditions are displayed in Fig. 4. First, we conducted a sanity check for the classification. In order to test if the 70% threshold resulted in comparably good representation of gaze patterns in both groups, the percentage values that exceeded the 70% threshold were retrieved per subject (e.g., if for a *gaze_target* type the two areas summed up to 80%, then the fit would have been 10% (= 80%–70%)). According to a Wilcoxon U test for non-normal data, these fits were comparable between groups (M_TD_ = 12.62% (SD_TD_ = 7.29)/ M_ASD_ = 12.54% (SD_ASD_ = 7.97); *U* = 2288, *p* = 0.919, *r* = 0.01).

**Table 3:** Summary statistic and group comparisons of dwell times (in ms) in groups and Regions of Interest (RoI)

	TD			ASD			Statistic	*p*	*effsize*
	*M*	*SD*	*Md*	*M*	*SD*	*Md*			
Gaze	1077	375	1091	580	487	465	252	< 0.001	0.485
Gesture	81	145	22	260	387	94	757	0.029	0.266
Target	278	210	271	332	206	293	642	0.439	0.095
Distractor	94	73	74	98	87	80	568	0.908	0.015
Upper body	156	192	75	307	277	259	797	0.007	0.326

Means, standard deviations, and medians of dwell times in groups (TD and ASD) and RoI (gaze, gesture, target, distractor, upper body). Results of Wilcoxon tests for group comparisons per RoI are reported

Two gaze types appeared as dominant in the TD group: 83.8% of TD participants were categorized as *gaze* or *gaze_target* types, irrespective of the IaPS condition. In the ASD group, only 36.76% of participants were categorized into these two types across both IaPS conditions. Overall, gaze types were more heterogeneous in the ASD group with increased numbers of gaze types that excluded the eye region of the character (i.e., RoI *gaze*; see Fig. 4). Furthermore, there were more within-subject gaze type changes in the ASD group, 61.8% of participants with ASD changed gaze types with IaPS conditions, whereas in the TD group, these were 44.1%, while from that 60.0% of changes were again between *gaze* and *gaze-target* types.

Extracting gaze coordinates per trial at the time point at which observers responded revealed again a preference for gaze in the TD group that was less pronounced in the ASD group, see Table 4. The gaze area was mostly fixated during keypresses in the TD group, whereas fixated areas during decision-making were more differentiated in the ASD group with largest percentages in the target RoI.

**Table 4:** Percentages of fixated Regions of Interest (RoI) at the time a decision was made by keypress

RoI	TD		ASD	
	*IaPS*_*TD*_	*IaPS*_*ASD*_	*IaPS*_*TD*_	*IaPS*_*ASD*_
Gaze	**47.58**	**56.46**	18.87	24.39
Point	4.78	6.35	14.07	12.88
Rbody	5.45	4.14	13.62	8.96
Target	35.80	27.15	**37.86**	**37.57**
Distractor	1.39	1.19	4.24	3.20
Random	5.00	4.71	11.33	13.00

All values in %. Largest values per group and IaPS condition (i.e., most fixated RoI during keypress) in bold

As visual information search strategies possibly affected decoding speeds, models predicting response times by gaze types were fitted for each group (see Table 2, Model 6). LRTs showed that gaze type explained a considerable amount of variance in the data in both groups (TD: *χ*^2^(1) = 141.07, *p* < 0.001; ASD: *χ*^2^(1) = 273.89, *p* < 0.001). Especially gaze types that excluded the characters eye region yielded fast response times, see Supplementary Fig. 2.

In sum, exploratory gaze analysis showed distinguishable visual information search behavior in both groups. TD individuals seemed to have favored the eye region of their virtual interaction partners whereas individuals with ASD presented with more diversified gaze types that add to the explanation of the group effect in decoding speeds.

### Impression formation

**Fig. 5:**
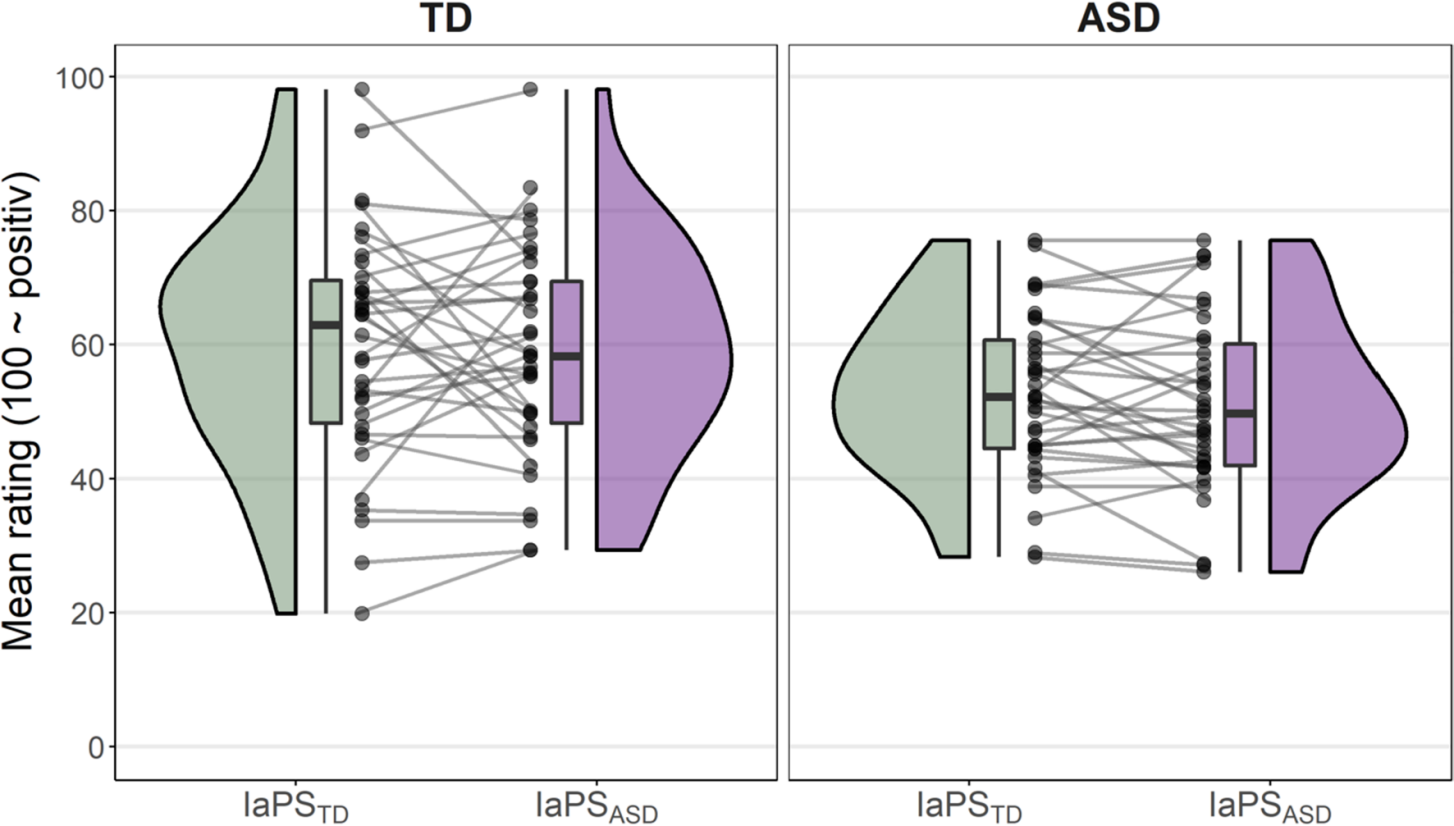
Mean post hoc character ratings per observer groups (TD, ASD) and IaPS conditions (IaPS_TD_, IaPS_ASD_). All character ratings were averaged per subject and IaPS condition. Mean ratings are displayed as clack dots. Connected dots display within-subject difference between IaPS conditions. Ratings were scaled so that “100” displays most positive impressions and “0” displays most negative impressions. Additionally, boxplots and density graphs show distributions of mean ratings in groups (TD left panel; ASD right panel) and IaPS conditions (IaPS_TD_ in green; IaPS_ASD_ in purple)

Aggregated mean ratings per subject in both IaPS conditions are depicted in Fig. 5. LRTs (Table 2, model 7) showed that the IaPS condition did not affect impression formation ratings above chance level (*χ*^2^(1) = 0.74, *p* = 0.390). TD individuals rated the virtual characters overall more positive than individuals with ASD (*χ*^2^(1) = 4.73, *p* = 0.030), however, this group effect did not interact with IaPS condition (*χ*^2^(1) = 0.10, *p* = 0.747).

## Discussion

Arguably, the intrapersonal temporal coordination of multimodal communication signals (e.g., gaze and gestures) is essential for communication efficiency. Given gaze–gesture coordination was shown to be particularly delayed and more variable in interactants with ASD [21, 27], the current study aimed to investigate the effects of group-specific intrapersonal synchrony (IaPS) of deictic gaze and pointing gestures. In a crossed design, we measured decoding speed, visual search behavior, and post-hoc impression formation in observers with and without ASD who interacted with virtual characters exhibiting group-specific couplings, or “in-group/out-group” patterns, of deictic gaze and pointing gestures.

Results showed that observing a virtual character prototypical of TD behavior (IaPS_TD_) yielded faster response times, hence, a higher communication efficiency compared to responding to a virtual character with ASD behavior (IaPS_ASD_). This effect was both found in observers with and without ASD and was even pronounced in observers with ASD, which contradicts an in-group advantage following the Double Empathy Hypothesis [65–67]. These results are consistent with findings that interpersonal synchrony is also reduced between individuals with ASD [16], so there is converging evidence against a within-group advantage for individuals with ASD at the temporal level of communication. Since gaze was always the first signal, as in the vast majority of real-life measures in Bloch et al. [21], and in accordance with literature of eye–hand coordination [68–71], a plausible outcome would have been that individuals would use gaze as a unimodal decision anchor. However, on a group-level, observers’ response times increased linearly with increasing gaze-gesture delays (Fig. 3B). There was no threshold of delays that made a qualitative difference but rather a graded effect of gaze–gesture delays. Again, the levels of increase contradicted the possibility that a particular gesture event (e.g., pointing peak) affected decoding speeds as a unimodal decision anchor. Instead, the data in both groups suggest an integration of gaze and pointing signals into one complex multimodal signal-unit during decoding. The size of the temporal delays thereby influenced decoding times, but to different degrees in observers with and without ASD.

We further investigated the possibility that the intrapersonal temporal coupling of multimodal signals could yield communicative effects that go beyond low-level decoding processes. In their study, de Marchena & Eigsti [20] found magnified delays between semantic aspects of speech and co-linguistic gestures in adolescents with ASD, which were associated with poorer ratings of communication quality by TD raters. In contrast, in the current study, there was no effect of the IaPS conditions on the impression that observers gained from their virtual interaction partners. As such, the global assumption that deviant temporal subtleties in multimodal nonverbal communication could influence higher-order impression formation was not supported by our data. These results further indicate that the differences between the two virtual characters’ effects on observers were not due to mere personal bias. Thus, the possibility that a negative overall impression of the virtual character influenced the efficiency of communication is not supported here. Especially since the subjects did not receive any information about the differences between the two characters before interacting with them in this rudimentary communication. It is therefore possible to infer that IaPS as a behavioral cue has no effect on impression formation on its own. Arguably, impression formation could rather be driven by more emotional behaviors, as in the stimulus material in Sasson et al. [24]. However, it should be noted that the high similarity in appearance of the two virtual characters and their highly reduced behavioral repertoire could have contributed to the absence of any such “personalized” effects. In addition, the interactive nature of the task was limited in that the virtual characters did not respond to the subjects’ choices. In this respect, the difference between the characters may have been too subtle to elicit differences in impression ratings.

Exploratory analysis of gaze behavior suggested that the group difference in decoding speeds was influenced by group-specific strategies of visual information retrieval. TD observers seemed to have deployed a rather homogeneous *eyes-focused* decoding strategy. This is in line with the assumption that a pointing gesture represents a spatially less ambiguous supplement to the primary and faster gaze signal with a potentially confirming feature [28, 29, 43]. It is also in line with the gaze being a special attractor for attention already from infancy onwards [72–76]. Furthermore, it should be taken into account that the eyes represent a spatially smaller signal, and thus focusing on the eyes is an efficient way of multimodal acquisition, since gestures could still be perceived peripherally with covert attention but eyes potentially could not. Thus, the gaze shift represented a fast directional signal, which, if performed at an appropriate temporal delay, could be supported or subsequently confirmed by a congruent pointing gesture. This temporal delay could be influenced by a suggested time window of about 350 ms opening after the gaze shift for the evaluation of social relevance and the intention to establish joint attention [77]. Interestingly, there was a sizeable TD subgroup who were classified as *gaze_target* types in the IaPS_TD_ condition, and switched to *gaze* types in the IaPS_ASD_ condition (see Fig. 4). This suggests that a temporally coherent pointing gesture, as produced by characters in the IaPS_TD_ condition, may have supported a shift of gaze to targets (i.e., responding to joint attention) and thus triadic attentional processes.

Decoding strategies turned out to be substantially different in observers with ASD. Individuals with ASD generally took longer to respond to the nonverbal communication. However, this group effect should be regarded under consideration of the gaze type analysis, which indicated a reduced focus on the partners’ gaze signal (i.e., more gaze types excluding RoI *gaze*). This is in line with other studies showing reduced eye region focus in observers with ASD [78–82]. As the gaze signal was the faster signal, a reduced focus on the partner’s eye region could at least partially explain the deceleration of response times. Notably, results showed that the increase of response times across different gaze-gesture delays was pronounced in individuals with ASD who were thus more affected by the variation in intrapersonal temporal alignment of multimodal signals. In analogy to the Double Empathy Hypothesis, one might have expected that individuals with ASD might decode nonverbal behavior faster and finally evaluate it better, which is consistent with the mode of production in ASD. However, the results in decoding times and visual information search strategies suggest here that observational behavior in ASD cannot be explained by an in-group effect. Taken together, the results indicate that individuals with ASD rather deployed strikingly *variable* decoding strategies. An increased involvement of the delayed pointing gesture region during visual search likely have contributed to the attenuation of decoding speeds. This group deviation is in line with ASD entailing atypicalities in attending to and processing gaze cues during joint attention episodes [30, 41, 42, 76, 83–85]. Possibly, such early peculiarities in gaze processing could explain the development of alternative strategies in multimodal communication, as exemplified in the highly variably decoding strategies shown in this study.

Using different strategies to decode multimodal nonverbal signals could potentially contribute to the understanding of reduced interpersonal synchrony in mixed dyads of individuals with and without ASD [16–18]. Koban et al. suggest that behavioral synchronization occurs due to the brain’s optimization principle [1]. Accordingly, the core functional principle is to reduce prediction errors through the matching of produced and perceived behavior. Deploying similar strategies to decode multimodal nonverbal behavior as shown for TD observers may probably result in more similar reciprocal response behavior, less prediction errors and ultimately more interpersonal synchrony. This contributes to timing differences in the production of multimodal nonverbal signals [21] that may further lead to a reciprocal mismatch of produced and perceived timed nonverbal behavior and thus to violations of priors in the sense of a Bayesian brain principle [1, 86–88].

Interpersonal synchrony has mostly been studied as a dependent variable, rather than as an independent variable [6]. Nevertheless, it is important to note that other factors influencing interpersonal synchrony have been studied or discussed (e.g., [89, 90]). Studies that investigate a direct relationship between IaPS and interpersonal synchrony are not yet available and should include these aspects as covariates.

### Limitations

As limitation aspects, it should be pointed out that the usage of virtual characters, besides the merits of high experimental control and standardization, limits the naturalism and ecological validity of the communication task. Thus, the generalizability of the present results to perceptual and response behavior in real-life scenarios is unclear.

Furthermore, the communication task should be clearly distinguished from naturalistic interaction tasks or tasks with a higher degree of interactivity that do not show the repetitive pattern of gaze and gestural behavior as implemented in this study.

Furthermore, impairments in executive functions in ASD could have added to response delays in the current study [91], yet could not explain differences between conditions, that were present in both observers with and without ASD.

The generalizability of the results is limited to adults with a F84.5 diagnosis according to ICD-10 and insofar it is unclear how the inclusion of a wider spectrum and age range would have affected the results.

The idea has been put forward that the majority or all psychiatric disorders might be associated with social impairments (e.g., depression or schizophrenia as cited in [92, 93]). While beyond the scope of the current study, future studies might investigate IaPS in a comparative fashion with other clinical groups to clarify specific coordination patterns.

## Conclusion

Characteristics of interactions between adults with and without ASD can be quantified by deviations in interpersonal synchronization [16, 17]. Differences in temporal communication patterns between multimodal signals *within* interactants (IaPS) could provide an explanation for such interpersonal deviations. To better understand interpersonal misalignment will require focusing on how interacting individuals process and produce temporal information during multimodal communication. The current results show that the multimodal communication of a virtual character is less efficient, if gaze and gesture are more detached and less integrated. This was the case for both observers with and without ASD. The contrast between a shared eyes-focused decoding strategy in TD observers versus heterogeneous decoding strategies in observers with ASD contribute to a reciprocal mismatch in the temporal dynamics of social interactions between individuals with and without ASD. Essentially, the current results place the symptom of reduced reciprocity in autism not in the individual, but directly in the interaction dynamics of a dyad.

## Supplementary Information

Below is the link to the electronic supplementary material.

Supplementary file1 (DOCX 973 KB)
We strive for an unbiased language in autism-related literature. We acknowledge different existent opinions on the use of ‘person-first’ versus ‘identity-first’ language [94–98]. Based on considerations in Tepest [98], we will use ‘person-first’ language throughout the manuscript.

## Data Availability

The conditions of our ethics approval do not permit public archiving of pseudonymized study data. Readers seeking access to the data should contact the corresponding author. Access to data that do not breach participant confidentiality will be granted to individuals for scientific purposes in accordance with ethical procedures governing the reuse of clinical data, including completion of a formal data sharing agreement. The scripts used for data acquisition and analysis are available from the corresponding author. The stimulus material cannot be published for licensing reasons, but can be requested from the corresponding author for scientific purposes.
