# Supplementary material for: Interacting with autistic virtual characters: intrapersonal synchrony of nonverbal behavior affects participants’ perception
Source: Eur Arch Psychiatry Clin Neurosci. 2024 Jan 25;274(7):1585–99. doi: 10.1007/s00406-023-01750-3 (PMC11422267; doi:10.1007/s00406-023-01750-3)
Supplement: Supplementary file 1 — Supplementary file1 (DOCX 973 KB) We strive for an unbiased language in autism-related literature. We acknowledge different existent opinions on the use of ‘person-first’ versus ‘identity-first’ language [94–98]. Based on considerations in Tepest [98], we will use ‘person-first’ language throughout the manuscript. [file 406_2023_1750_MOESM1_ESM.docx]

**[SUPPLEMENTARY MATERIAL]**

**Interacting with autistic virtual characters: Timing of nonverbal behavior affects participants' perception**

Carola Bloch*, Ralf Tepest, Sevim Koeroglu, Kyra Feikes, Mathis Jording, Kai Vogeley^**^, Christine M. Falter-Wagner^**^

* Corresponding author (carola.bloch@med.uni-muenchen.de)

** Christine M. Falter-Wagner and Kai Vogeley share last-authorship

**Supplementary Material 1.** Pilot study

Because one hypothesis addressed the effects of IaPS production on subsequent impression formation, we sought to prevent biased judgments due to possible differences in the general appearance of the virtual characters. Thus, we conducted a pilot study to select two characters that were judged most similar based on their bodily appearance. For that purpose, ten virtual characters were created in Autodesk ® Character Generator. In an online assessment, 32 individuals (23 identified as female, 9 as male; aged M = 38.75 (SD = 12.56)) rated each character displayed as frontal profile pictures on various dimensions related to age, gender, personality traits, emotional status, and uncanny valley (Ho & MacDorman, 2017). These dimensions were: age; gender; attractiveness; friendliness; aggressiveness; trustworthiness; conscientiousness; interest; emotional stability; sadness; compatibility; sociableness; dominance; extroversion; intelligence; neutrality; eeriness; realism; strangeness. The assessment revealed that two characters could be selected from the initial set that did not differ above chance-level on any of the dimensions mentioned above except age (see **Supplementary Table 1**).

**Supplementary Material 2.** Animation

For the creation of the virtual scenario in this study, a virtual room was created to resemble the experimental setup from the previous study. A virtual table was created and the characters were positioned opposite to the observer, sitting at the table with their right arm resting on the table and their gaze directed ahead. Scenes were rendered from a camera position similar to the perspective of the interaction partner in the previous study. Thus, a straight gaze direction of the characters appeared as eye-contact in the video stimuli that were used in the current study.

Spatial parameters (i.e., amplitudes) of the animated characters’ pointing gesture (distance between the point at which the pointing gesture started and the one at which it lingered: left = 17 cm; right = 25 cm) and saccade (17° in visual angle horizontally per side) were empirically informed by the previous study. Temporal parameters included eye-contact duration, gaze latency, gaze shift duration, gesture duration, and the temporal delay between gaze and pointing gesture onsets. The aforementioned delay was varied as the experimental manipulation of produced IaPS (see section **2.4. Virtual interaction task design** in main text for exact temporal parameters). All other spatial and temporal parameters were kept constant for both characters as there were no group differences on these dimensions in the previous study (Bloch et al., 2022). The temporal parameters were implemented by keyframe animation, in which the start- and end-points of gaze and gesture events were set on a shared timeline in 40 fps resolution (25 ms per frame).

**Supplementary Material 3.** Gaze type classification

Gaze types were defined as the combination of regions of interest (RoIs), ordered by relative dwell times, that cumulated to 70% of total dwell times. For example, if a participant fixated primarily on the characters eyes (i.e., highest aggregated percentage value for RoI_GAZE_ of $\geq$ 70%) then this person would have been classified as the gaze type *gaze*. If the percentage value for RoI_GAZE_ would have been < 70%, then the RoI with the second highest relative dwell time (e.g., RoI_POINT_) would have been added to the gaze type and this person would have been classified as *gaze_gesture*. If the aggregated dwell time percentage was still below 70% the third RoI would have been added and so forth. For complexity reduction, the order of gaze areas in the gaze types names do not reflect the descending order of percentage sizes, instead names of gaze areas were ordered alphabetically in gaze types designations. For example two cases with i) first RoI_GAZE_ (48 %) and second RoI_POINT_ (30 %) ii) first RoI_POINT_ (61 %) and second RoI_GAZE_ (22%) would have resulted in the gaze type *gaze_gesture*. Thus, gaze types provided insight into the information-seeking strategies of individuals and if the strategies change with group-specific expression of IaPS (i.e., IaPS_ASD_ vs. IaPS_TD_). Additionally to dwell times in RoI, the RoI at which participants’ gaze was located at making a decision (i.e., time of key press) was extracted from the gaze data.

**Supplementary Table 1.** Virtual character ratings from pilot study

| **Item** | **Character A** | **Character B** | **Comparison** |
| --- | --- | --- | --- |
| *Gender* | 2.0 (4.2) | 3.0 (6.9) | *U* = 517, *p* = .935 |
| *Age* | 30.8 (5.0) | 27.4 (6.0) | *U* = 695, *p* = .014 |
| *Attractiveness* | 3.7 (1.0) | 3.4 (1.0) | *U* = 565, *p* = .453 |
| *Friendliness* | 3.7 (0.8) | 3.8 (0.9) | *U* = 496, *p* = .813 |
| *Aggressiveness* | 2.4 (1.2) | 2.6 (1.2) | *U* = 465, *p* = .520 |
| *Trustworthiness* | 3.8 (0.7) | 3.6 (0.9) | *U* = 562, *p* = .477 |
| *Conscientiousness* | 3.9 (0.8) | 3.8 (0.8) | *U* = 545, *p* = .620 |
| *Interest* | 3.9 (0.8) | 3.7 (0.9) | *U* = 573, *p* = .371 |
| *Emotional stability* | 3.9 (0.8) | 3.8 (0.8) | *U* = 528, *p* = .818 |
| *Sadness* | 2.6 (1.2) | 2.6 (1.1) | *U* = 526, *p* = .851 |
| *Compatibility* | 4.0 (0.9) | 3.8 (0.8) | *U* = 574, *p* = .363 |
| *Sociableness* | 3.9 (1.0) | 3.9 (0.8) | *U* = 521, *p* = .903 |
| *Dominance* | 3.2 (1.2) | 3.1 (1.2) | *U* = 522, *p* = .890 |
| *Extroversion* | 3.2 (1.2) | 3.6 (1.0) | *U* = 424, *p* = .221 |
| *Intelligence* | 3.8 (0.9) | 3.8 (0.8) | *U* = 507, *p* = .947 |
| *Neutrality* | 3.5 (0.8) | 3.6 (0.8) | *U* = 448, *p* = .354 |
| *Eeriness* | 2.5 (1.5) | 2.7 (1.4) | *U* = 468, *p* = .549 |
| *Realism* | 3.9 (1.2) | 3.7 (1.1) | *U* = 560, *p* = .503 |
| *Strangeness* | 2.9 (1.1) | 3.1 (1.4) | *U* = 464, *p* = .514 |

*Note*. Mean ratings and standard deviation per character. Shapiro-Wilk tests indicated that assumption of normal distribution was violated. Wilcoxon U-tests for non-normal data are reported. No correction for multiple testing applied.

**Supplementary Table 2.** Fixed effects estimates with standard errors and significance tests

| *Predictors* | *Estimates* | *CI* | *p* |
| --- | --- | --- | --- |
| **group** | **70.83** | **22.17 – 119.49** | **.004** |
| **condition** | **35.00** | **17.00 – 53.01** | **< .001** |
| **block** | **-30.76** | **-38.66 - -22.87** | **< .001** |
| **group*condition** | **18.15** | **0.15 – 36.16** | **.048** |
| group*block | -0.81 | -8.71 – 7.08 | .840 |
| condition*block | 0.30 | -8.48 – 9.07 | .947 |
| group*condition*block | -2.17 | -10.95 – 6.60 | .628 |

*Note*. Output summary of fixed effects of linear mixed effects Model 4 (Table 2 in main article) predicting response times in ms.

**Supplementary Table 3.** Fixed effects estimates with standard errors and significance tests

| *Predictors* | *Estimates* | *CI* | *p* |
| --- | --- | --- | --- |
| **group** | **70.24** | **20.84 – 119.63** | **.005** |
| **condition** | **35.20** | **17.04 – 53.35** | **< .001** |
| correction | 7.96 | -41.43 – 57.35 | .752 |
| group*condition | 17.38 | -0.77 – 35.54 | .061 |
| group*correction | 12.09 | -37.30 – 61.48 | .631 |
| condition*correction | -7.75 | -25.91 – 10.40 | .402 |
| group*condition*correction | 1.07 | -17.08 – 19.22 | .908 |

*Note*. Output summary of fixed effects of linear mixed effects Model 5 (Table 2 in main article) predicting response times in ms.

**Supplementary Fig. 1: Response times in observer groups across experimental blocks.** **(A)** Response times averaged across experimental blocks for observer groups (TD in yellow; ASD in blue). Errorbars represent standard errors of the means. IaPS conditions are depicted as solid (IaPS_ASD_) and dashed (IaPS_TD_) lines. **(B)** Response times averaged over gaze-gesture delay conditions, colored by experimental blocks (1-3) and line types represent observer groups (ASD group as solid lines; TD group as dashed lines).


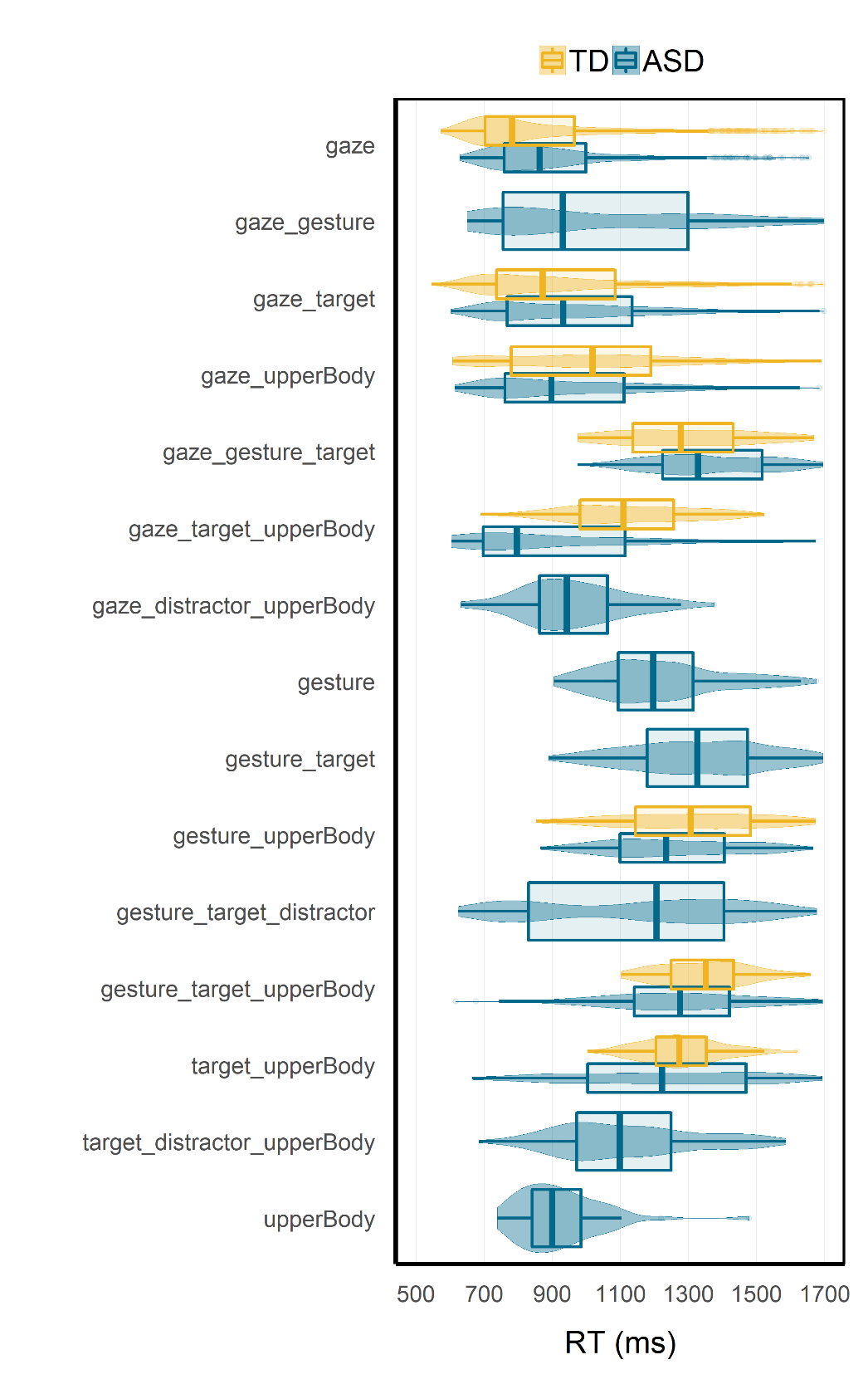


**Supplementary Fig. 2: Response times (ms) in gaze types (y-axis) and observer groups.** Violin plots per observer group (TD group in yellow; ASD group in blue) with overlaid boxplots depict response times per gaze type. Some gaze types were only present in the ASD observer group so that only response times for this group are displayed.
